# Crystal structure of 5-fluoro-2-(3-fluoro­phen­yl)-3-methyl­sulfinyl-1-benzo­furan

**DOI:** 10.1107/S160053681402251X

**Published:** 2014-10-18

**Authors:** Hong Dae Choi, Uk Lee

**Affiliations:** a Department of Chemistry, Dongeui University, San 24 Kaya-dong, Busanjin-gu, Busan 614-714, Republic of Korea; b Department of Chemistry, Pukyong National University, 599-1 Daeyeon 3-dong, Nam-gu, Busan 608-737, Republic of Korea

**Keywords:** crystal structure, benzo­furan, 3-fluoro­phen­yl, π–π inter­actions.

## Abstract

In the title compound, C_15_H_10_F_2_O_2_S, the dihedral angle between the planes of the benzo­furan ring system [r.m.s. deviation = 0.015 (1) Å] and the 3-fluoro­phenyl ring is 26.60 (5)°. In the crystal, mol­ecules are linked by C—H⋯O and C—H⋯F hydrogen bonds, and by π–π inter­actions between the benzo­furan rings of inversion-related mol­ecules [centroid(benzene)–centroid(furan) distance = 3.819 (2) Å], forming a three-dimensional network.

## Related literature   

For a related structure and background to benzo­furan derivatives, see: Choi & Lee (2014 ▶). For further synthetic details, see: Choi *et al.* (1999 ▶).
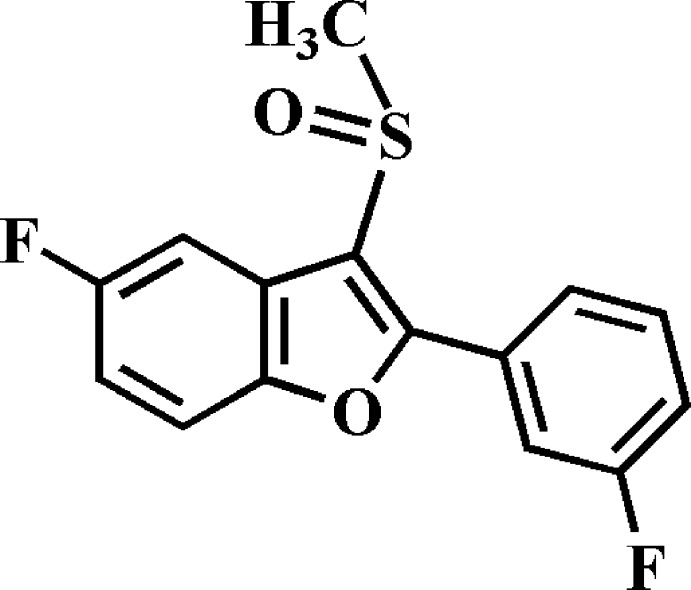


## Experimental   

### Crystal data  

C_15_H_10_F_2_O_2_S
*M* *_r_* = 292.29
Monoclinic, 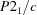

*a* = 8.4826 (2) Å
*b* = 16.6307 (4) Å
*c* = 9.7493 (2) Å
β = 113.756 (1)°
*V* = 1258.81 (5) Å^3^
*Z* = 4
Mo *K*α radiation
μ = 0.28 mm^−1^
*T* = 173 K
0.62 × 0.55 × 0.42 mm

### Data collection  

Bruker SMART APEXII CCD diffractometer
Absorption correction: multi-scan (*SADABS*; Bruker, 2009 ▶) *T*_min_ = 0.846, *T*_max_ = 0.892
12021 measured reflections
3111 independent reflections
2736 reflections with *I* > 2σ(*I*)
*R*_int_ = 0.027

### Refinement  

*R*[*F* ^2^ > 2σ(*F* ^2^)] = 0.036
*wR*(*F* ^2^) = 0.098
*S* = 1.03
3111 reflections
182 parameters
H-atom parameters constrained
Δρ_max_ = 0.41 e Å^−3^
Δρ_min_ = −0.35 e Å^−3^

### 

Data collection: *APEX2* (Bruker, 2009 ▶); cell refinement: *SAINT* (Bruker, 2009 ▶); data reduction: *SAINT*; program(s) used to solve structure: *SHELXS97* (Sheldrick, 2008 ▶); program(s) used to refine structure: *SHELXL97* (Sheldrick, 2008 ▶); molecular graphics: *ORTEP-3 for Windows* (Farrugia, 2012 ▶) and *DIAMOND* (Brandenburg, 1998 ▶); software used to prepare material for publication: *SHELXL97*.

## Supplementary Material

Crystal structure: contains datablock(s) I. DOI: 10.1107/S160053681402251X/fy2119sup1.cif

Structure factors: contains datablock(s) I. DOI: 10.1107/S160053681402251X/fy2119Isup2.hkl

Supporting information file. DOI: 10.1107/S160053681402251X/fy2119Isup3.cml

**Fig. 1.**
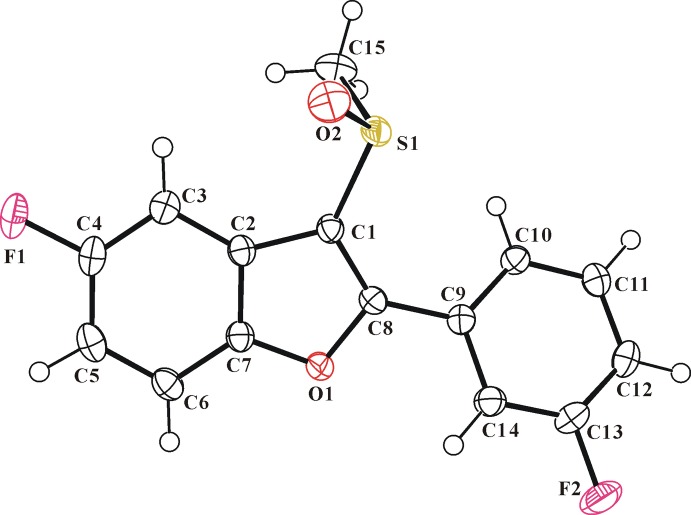
The molecular structure of the title compound with the atom numbering scheme. Displacement ellipsoids are drawn at the 50% probability level. H atoms are presented as small spheres of arbitrary radius.

. DOI: 10.1107/S160053681402251X/fy2119fig1.tif
The mol­ecular structure of the title compound with the atom numbering scheme. Displacement ellipsoids are drawn at the 50% probability level. H atoms are presented as small spheres of arbitrary radius.

**Fig. 2.**
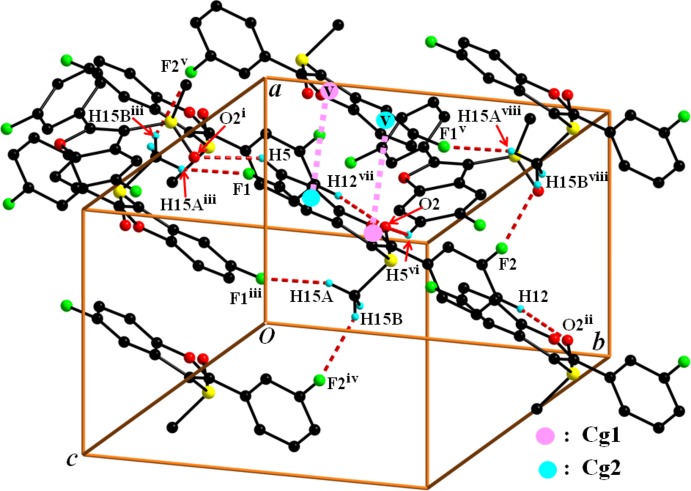
A view of the C—H···O, C—H···F and π–π interactions (dotted lines) in the crystal structure of the title compound. H atoms not participating in hydrogen-bonding were omitted for clarity. [Symmetry codes: (i) - x + 2, y -1/2, - z + 3/2; (ii) x - 1, y, z - 1; (iii) - x + 2, - y + 1, - z + 2; (iv) x, y, z + 1; (v) - x + 2, - y + 1, - z + 1; (vi) - x + 2, y + 1/2, - z + 3/2; (vii) x + 1, y, z + 1; (viii) - x + 2, y + 1/2, - z + 3/2.]

Click here for additional data file.x y z x y z x y z x y z x y z x y z x y z x y z . DOI: 10.1107/S160053681402251X/fy2119fig2.tif
A view of the C—H⋯O, C—H⋯F and π–π inter­actions (dotted lines) in the crystal structure of the title compound. H atoms not participating in hydrogen-bonding were omitted for clarity. [Symmetry codes: (i) − *x* + 2, *y* − 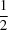, − *z* + 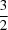; (ii) *x* − 1, *y*, *z* − 1; (iii) − *x* + 2, − *y* + 1, − *z* + 2; (iv) *x*, *y*, *z* + 1; (v) − *x* + 2, − *y* + 1, − *z* + 1; (vi) − *x* + 2, *y* + 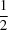, − *z* + 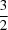; (vii) *x* + 1, *y*, *z* + 1; (viii) − *x* + 2, *y* + 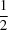, − *z* + 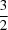.]

CCDC reference: 1029041

Additional supporting information:  crystallographic information; 3D view; checkCIF report

## Figures and Tables

**Table 1:** Hydrogen-bond geometry (, )

*D*H*A*	*D*H	H*A*	*D* *A*	*D*H*A*
C5H5O2^i^	0.95	2.45	3.2612(18)	143
C12H12O2^ii^	0.95	2.42	3.3361(19)	161
C15H15*A*F1^iii^	0.98	2.54	3.409(2)	147
C15H15*B*F2^iv^	0.98	2.55	3.163(2)	121

Symmetry codes: (i) 
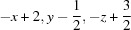
; (ii) 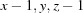; (iii) 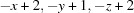; (iv) 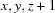.

## supplementary crystallographic information

### S1. Comment

As a part of our continuing program for benzofuran derivatives (Choi & Lee, 2014), we report herein on the crystal structure of the title compound.

In the title molecule (Fig. 1), the benzofuran unit is essentially planar, with a mean deviation of 0.015 (1) Å from the least-squares plane defined by the nine constituent atoms. The 3-fluorophenyl ring is essentially planar, with a mean deviation of 0.009 (1) Å from the least-squares plane defined by the six constituent atoms. The dihedral angle formed by the benzofuran ring and the 3-fluorophenyl ring is 26.60 (5)°. In the crystal structure (Fig. 2), molecules are linked by C—H···O and C—H···F hydrogen bonds (Table 1), and by π–π interactions between the benzene and furan rings of neighbouring molecules, with a *Cg*1···*Cg*2^v^ distance of 3.819 (2) Å and an interplanar distance of 3.283 (2) Å resulting in a slippage of 1.951 (2) Å (*Cg*1 and *Cg*2 are the centroids of the C2–C7 benzene ring and C1/C2/C7/O1/C8 furan ring, respectively), forming a three-dimensional network.

### S2. Experimental

The starting material 5-fluoro-2-(3-fluorophenyl)-3-methylsulfanyl-1-benzofuran was prepared by literature method (Choi *et al.*, 1999). 3-Chloroperoxybenzoic acid (77%, 269 mg, 1.2 mmol) was added in small portions to a stirred solution of 5-fluoro-2-(3-fluorophenyl)-3-methylsulfanyl-1-benzofuran (304 mg, 1.1 mmol) in dichloromethane (30 ml) at 273 K. After being stirred at room temperature for 8h, the mixture was washed with saturated sodium bicarbonate solution (2 × 10 ml) and the organic layer was separated, dried over magnesium sulfate, filtered and concentrated at reduced pressure. The residue was purified by column chromatography (hexane–ethyl acetate, 1:2 *v*/*v*) to afford the title compound as a colorless solid [yield 71% (207 mg); m.p. 459–460 K; *R*_f_ = 0.48 (hexane–ethyl acetate, 1:2 *v*/*v*)]. Single crystals suitable for X-ray diffraction were prepared by slow evaporation of a solution of the title compound (24 mg) in acetone (20 ml) at room temperature.

### S3. Refinement

All H atoms were positioned geometrically and refined using a riding model, with C—H = 0.95 Å for aryl, and 0.98 Å for methyl H atoms, respectively. *U*_iso_ (H) = 1.2*U*_eq_ (C) for aryl and 1.5*U*_eq_ (C) for methyl H atoms. The rotations of methyl groups were optimized using the SHELXL-97 command AFIX 137 (Sheldrick, 2008).

### Crystal data

**Table:**

C_15_H_10_F_2_O_2_S	*F*(000) = 600
*M**_r_* = 292.29	*D*_x_ = 1.542 Mg m^−^^3^
Monoclinic, *P*2_1_/*c*	Melting point = 460–459 K
Hall symbol: -P 2ybc	Mo *K*α radiation, λ = 0.71073 Å
*a* = 8.4826 (2) Å	Cell parameters from 5007 reflections
*b* = 16.6307 (4) Å	θ = 2.5–28.3°
*c* = 9.7493 (2) Å	µ = 0.28 mm^−^^1^
β = 113.756 (1)°	*T* = 173 K
*V* = 1258.81 (5) Å^3^	Block, colourless
*Z* = 4	0.62 × 0.55 × 0.42 mm

### Data collection

**Table:**

Bruker SMART APEXII CCD diffractometer	3111 independent reflections
Radiation source: rotating anode	2736 reflections with *I* > 2σ(*I*)
Graphite multilayer monochromator	*R*_int_ = 0.027
Detector resolution: 10.0 pixels mm^-1^	θ_max_ = 28.3°, θ_min_ = 2.5°
φ and ω scans	*h* = −11→11
Absorption correction: multi-scan (*SADABS*; Bruker, 2009)	*k* = −18→22
*T*_min_ = 0.846, *T*_max_ = 0.892	*l* = −12→12
12021 measured reflections	

### Refinement

**Table:**

Refinement on *F*^2^	Primary atom site location: structure-invariant direct methods
Least-squares matrix: full	Secondary atom site location: difference Fourier map
*R*[*F*^2^ > 2σ(*F*^2^)] = 0.036	Hydrogen site location: difference Fourier map
*wR*(*F*^2^) = 0.098	H-atom parameters constrained
*S* = 1.03	*w* = 1/[σ^2^(*F*_o_^2^) + (0.0527*P*)^2^ + 0.4861*P*] where *P* = (*F*_o_^2^ + 2*F*_c_^2^)/3
3111 reflections	(Δ/σ)_max_ = 0.001
182 parameters	Δρ_max_ = 0.41 e Å^−^^3^
0 restraints	Δρ_min_ = −0.35 e Å^−^^3^

### Special details

**Table:**

Experimental. ^1^H NMR (δ p.p.m., CDCl_3_, 400 Hz): 7.91 (dd, J =8.56 and 2.40 Hz, 1H), 7.47-7.64 (m, 4H), 7.13-7.22 (m, 2H), 3.11 (s, 3H).
Geometry. All esds (except the esd in the dihedral angle between two l.s. planes) are estimated using the full covariance matrix. The cell esds are taken into account individually in the estimation of esds in distances, angles and torsion angles; correlations between esds in cell parameters are only used when they are defined by crystal symmetry. An approximate (isotropic) treatment of cell esds is used for estimating esds involving l.s. planes.
Refinement. Refinement of F^2^ against ALL reflections. The weighted R-factor wR and goodness of fit S are based on F^2^, conventional R-factors R are based on F, with F set to zero for negative F^2^. The threshold expression of F^2^ > 2sigma(F^2^) is used only for calculating R-factors(gt) etc. and is not relevant to the choice of reflections for refinement. R-factors based on F^2^ are statistically about twice as large as those based on F, and R- factors based on ALL data will be even larger.

### Fractional atomic coordinates and isotropic or equivalent isotropic displacement parameters (Å^2^)

**Table:**

	*x*	*y*	*z*	*U*_iso_*/*U*_eq_	
S1	0.68157 (5)	0.69175 (2)	0.61814 (4)	0.02500 (11)	
F1	1.15704 (13)	0.43458 (6)	0.91143 (11)	0.0407 (3)	
F2	0.31417 (14)	0.61447 (8)	−0.13611 (11)	0.0455 (3)	
O1	0.66160 (13)	0.50305 (6)	0.36646 (11)	0.0228 (2)	
O2	0.86399 (15)	0.71766 (7)	0.69804 (14)	0.0380 (3)	
C1	0.68851 (17)	0.59646 (8)	0.54180 (15)	0.0206 (3)	
C2	0.81117 (17)	0.53330 (8)	0.61313 (16)	0.0213 (3)	
C3	0.93765 (18)	0.51943 (9)	0.75628 (16)	0.0257 (3)	
H3	0.9568	0.5554	0.8371	0.031*	
C4	1.03265 (19)	0.45056 (9)	0.77310 (17)	0.0284 (3)	
C5	1.0128 (2)	0.39587 (9)	0.65947 (19)	0.0288 (3)	
H5	1.0839	0.3494	0.6794	0.035*	
C6	0.88870 (19)	0.40976 (8)	0.51726 (18)	0.0263 (3)	
H6	0.8720	0.3742	0.4364	0.032*	
C7	0.79001 (17)	0.47831 (8)	0.49901 (16)	0.0220 (3)	
C8	0.60314 (17)	0.57525 (8)	0.39537 (15)	0.0207 (3)	
C9	0.46688 (17)	0.61298 (8)	0.26667 (15)	0.0211 (3)	
C10	0.34606 (18)	0.66392 (8)	0.28439 (16)	0.0231 (3)	
H10	0.3529	0.6755	0.3821	0.028*	
C11	0.21572 (19)	0.69790 (9)	0.16032 (17)	0.0263 (3)	
H11	0.1338	0.7324	0.1737	0.032*	
C12	0.20422 (19)	0.68181 (9)	0.01715 (17)	0.0280 (3)	
H12	0.1159	0.7049	−0.0685	0.034*	
C13	0.3254 (2)	0.63115 (10)	0.00347 (16)	0.0287 (3)	
C14	0.45608 (19)	0.59613 (9)	0.12251 (17)	0.0262 (3)	
H14	0.5368	0.5615	0.1076	0.031*	
C15	0.6103 (2)	0.66132 (11)	0.7597 (2)	0.0377 (4)	
H15A	0.6903	0.6213	0.8250	0.057*	
H15B	0.4949	0.6378	0.7123	0.057*	
H15C	0.6068	0.7082	0.8193	0.057*	

### Atomic displacement parameters (Å^2^)

**Table:**

	*U*^11^	*U*^22^	*U*^33^	*U*^12^	*U*^13^	*U*^23^
S1	0.0261 (2)	0.01759 (17)	0.02614 (19)	−0.00056 (12)	0.00519 (15)	−0.00347 (13)
F1	0.0340 (5)	0.0452 (6)	0.0317 (5)	0.0116 (4)	0.0017 (4)	0.0127 (4)
F2	0.0443 (6)	0.0705 (8)	0.0194 (5)	0.0076 (5)	0.0105 (4)	−0.0019 (4)
O1	0.0240 (5)	0.0197 (5)	0.0223 (5)	0.0023 (4)	0.0069 (4)	−0.0015 (4)
O2	0.0311 (6)	0.0311 (6)	0.0427 (7)	−0.0107 (5)	0.0055 (5)	−0.0085 (5)
C1	0.0204 (6)	0.0183 (6)	0.0215 (6)	0.0002 (5)	0.0068 (5)	0.0003 (5)
C2	0.0202 (6)	0.0192 (6)	0.0240 (7)	−0.0003 (5)	0.0086 (5)	0.0019 (5)
C3	0.0245 (7)	0.0262 (7)	0.0244 (7)	−0.0006 (6)	0.0077 (6)	0.0020 (5)
C4	0.0225 (7)	0.0312 (8)	0.0276 (7)	0.0027 (6)	0.0061 (6)	0.0099 (6)
C5	0.0258 (7)	0.0230 (7)	0.0395 (9)	0.0049 (5)	0.0152 (7)	0.0085 (6)
C6	0.0291 (7)	0.0198 (6)	0.0328 (8)	0.0017 (5)	0.0153 (6)	0.0017 (5)
C7	0.0221 (6)	0.0197 (6)	0.0238 (7)	−0.0004 (5)	0.0087 (6)	0.0021 (5)
C8	0.0207 (6)	0.0187 (6)	0.0229 (7)	0.0001 (5)	0.0090 (5)	−0.0007 (5)
C9	0.0188 (6)	0.0200 (6)	0.0224 (7)	−0.0024 (5)	0.0061 (5)	−0.0002 (5)
C10	0.0222 (6)	0.0252 (7)	0.0214 (6)	−0.0003 (5)	0.0081 (5)	−0.0001 (5)
C11	0.0228 (7)	0.0245 (7)	0.0303 (7)	0.0021 (5)	0.0092 (6)	0.0023 (6)
C12	0.0227 (7)	0.0314 (8)	0.0238 (7)	−0.0003 (6)	0.0030 (6)	0.0062 (6)
C13	0.0285 (7)	0.0371 (8)	0.0190 (7)	−0.0035 (6)	0.0081 (6)	−0.0009 (6)
C14	0.0243 (7)	0.0301 (7)	0.0240 (7)	0.0014 (5)	0.0095 (6)	−0.0021 (6)
C15	0.0439 (10)	0.0383 (9)	0.0378 (9)	0.0002 (7)	0.0237 (8)	−0.0091 (7)

### Geometric parameters (Å, º)

**Table:**

S1—O2	1.4885 (12)	C6—C7	1.3828 (19)
S1—C1	1.7619 (14)	C6—H6	0.9500
S1—C15	1.7880 (17)	C8—C9	1.4614 (19)
F1—C4	1.3624 (17)	C9—C10	1.3930 (19)
F2—C13	1.3540 (17)	C9—C14	1.399 (2)
O1—C8	1.3710 (16)	C10—C11	1.388 (2)
O1—C7	1.3754 (17)	C10—H10	0.9500
C1—C8	1.3616 (19)	C11—C12	1.385 (2)
C1—C2	1.4444 (19)	C11—H11	0.9500
C2—C7	1.3949 (19)	C12—C13	1.377 (2)
C2—C3	1.395 (2)	C12—H12	0.9500
C3—C4	1.372 (2)	C13—C14	1.370 (2)
C3—H3	0.9500	C14—H14	0.9500
C4—C5	1.390 (2)	C15—H15A	0.9800
C5—C6	1.381 (2)	C15—H15B	0.9800
C5—H5	0.9500	C15—H15C	0.9800
			
O2—S1—C1	106.07 (7)	C1—C8—C9	133.75 (12)
O2—S1—C15	106.34 (8)	O1—C8—C9	115.22 (11)
C1—S1—C15	98.65 (7)	C10—C9—C14	119.50 (13)
C8—O1—C7	106.50 (10)	C10—C9—C8	121.44 (12)
C8—C1—C2	106.99 (12)	C14—C9—C8	119.04 (12)
C8—C1—S1	125.57 (11)	C11—C10—C9	120.46 (13)
C2—C1—S1	126.22 (11)	C11—C10—H10	119.8
C7—C2—C3	119.31 (13)	C9—C10—H10	119.8
C7—C2—C1	104.86 (12)	C12—C11—C10	120.49 (13)
C3—C2—C1	135.77 (13)	C12—C11—H11	119.8
C4—C3—C2	115.84 (14)	C10—C11—H11	119.8
C4—C3—H3	122.1	C13—C12—C11	117.62 (13)
C2—C3—H3	122.1	C13—C12—H12	121.2
F1—C4—C3	117.68 (14)	C11—C12—H12	121.2
F1—C4—C5	117.26 (13)	F2—C13—C14	118.00 (14)
C3—C4—C5	125.05 (14)	F2—C13—C12	118.02 (14)
C6—C5—C4	119.27 (13)	C14—C13—C12	123.98 (14)
C6—C5—H5	120.4	C13—C14—C9	117.95 (13)
C4—C5—H5	120.4	C13—C14—H14	121.0
C5—C6—C7	116.38 (14)	C9—C14—H14	121.0
C5—C6—H6	121.8	S1—C15—H15A	109.5
C7—C6—H6	121.8	S1—C15—H15B	109.5
O1—C7—C6	125.23 (13)	H15A—C15—H15B	109.5
O1—C7—C2	110.61 (11)	S1—C15—H15C	109.5
C6—C7—C2	124.14 (14)	H15A—C15—H15C	109.5
C1—C8—O1	111.03 (12)	H15B—C15—H15C	109.5
			
O2—S1—C1—C8	−126.44 (13)	C1—C2—C7—C6	176.94 (13)
C15—S1—C1—C8	123.68 (13)	C2—C1—C8—O1	−0.09 (15)
O2—S1—C1—C2	39.24 (14)	S1—C1—C8—O1	167.87 (9)
C15—S1—C1—C2	−70.64 (14)	C2—C1—C8—C9	−179.62 (14)
C8—C1—C2—C7	0.90 (15)	S1—C1—C8—C9	−11.7 (2)
S1—C1—C2—C7	−166.96 (10)	C7—O1—C8—C1	−0.78 (15)
C8—C1—C2—C3	177.95 (15)	C7—O1—C8—C9	178.85 (11)
S1—C1—C2—C3	10.1 (2)	C1—C8—C9—C10	−28.6 (2)
C7—C2—C3—C4	−0.32 (19)	O1—C8—C9—C10	151.91 (12)
C1—C2—C3—C4	−177.04 (15)	C1—C8—C9—C14	152.70 (16)
C2—C3—C4—F1	−179.70 (12)	O1—C8—C9—C14	−26.82 (18)
C2—C3—C4—C5	0.8 (2)	C14—C9—C10—C11	0.0 (2)
F1—C4—C5—C6	−179.77 (13)	C8—C9—C10—C11	−178.75 (13)
C3—C4—C5—C6	−0.3 (2)	C9—C10—C11—C12	−0.2 (2)
C4—C5—C6—C7	−0.7 (2)	C10—C11—C12—C13	0.3 (2)
C8—O1—C7—C6	−176.95 (13)	C11—C12—C13—F2	179.38 (13)
C8—O1—C7—C2	1.38 (14)	C11—C12—C13—C14	−0.2 (2)
C5—C6—C7—O1	179.33 (13)	F2—C13—C14—C9	−179.58 (13)
C5—C6—C7—C2	1.2 (2)	C12—C13—C14—C9	0.0 (2)
C3—C2—C7—O1	−179.05 (11)	C10—C9—C14—C13	0.1 (2)
C1—C2—C7—O1	−1.41 (15)	C8—C9—C14—C13	178.89 (13)
C3—C2—C7—C6	−0.7 (2)		

### Hydrogen-bond geometry (Å, º)

**Table:**

*D*—H···*A*	*D*—H	H···*A*	*D*···*A*	*D*—H···*A*
C5—H5···O2^i^	0.95	2.45	3.2612 (18)	143
C12—H12···O2^ii^	0.95	2.42	3.3361 (19)	161
C15—H15*A*···F1^iii^	0.98	2.54	3.409 (2)	147
C15—H15*B*···F2^iv^	0.98	2.55	3.163 (2)	121

Symmetry codes: (i) −*x*+2, *y*−1/2, −*z*+3/2; (ii) *x*−1, *y*, *z*−1; (iii) −*x*+2, −*y*+1, −*z*+2; (iv) *x*, *y*, *z*+1.
