# Supplementary figures and images for: Crystal structure of 5-fluoro-2-(3-fluoro­phen­yl)-3-methyl­sulfinyl-1-benzo­furan
Source: Acta Crystallogr Sect E Struct Rep Online. 2014 Oct 18;70(Pt 11):o1168. doi: 10.1107/S160053681402251X (PMC4257342; doi:10.1107/S160053681402251X)

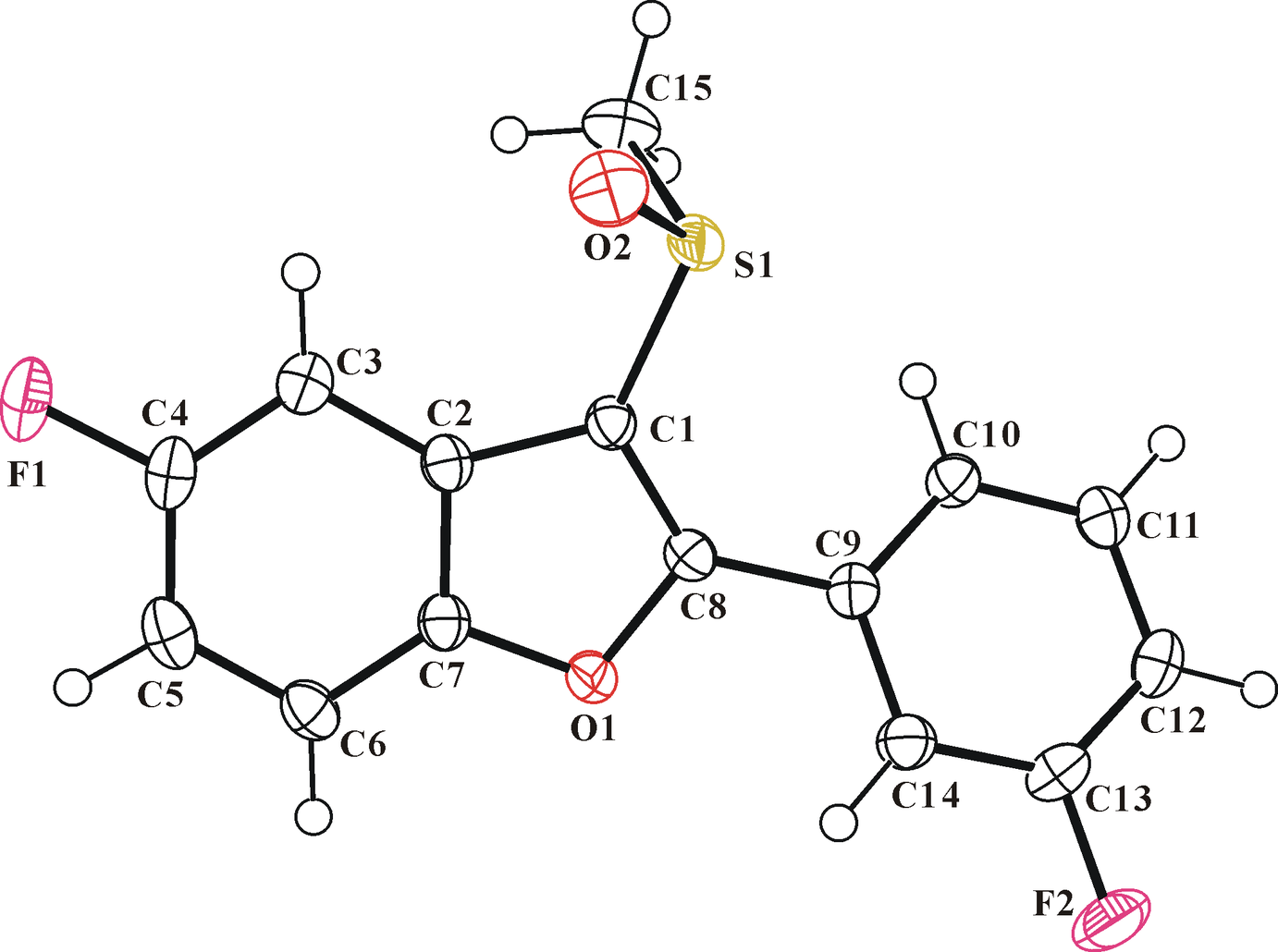

Supplement: Supplementary file 4 [file e-70-o1168-fig1.tif]

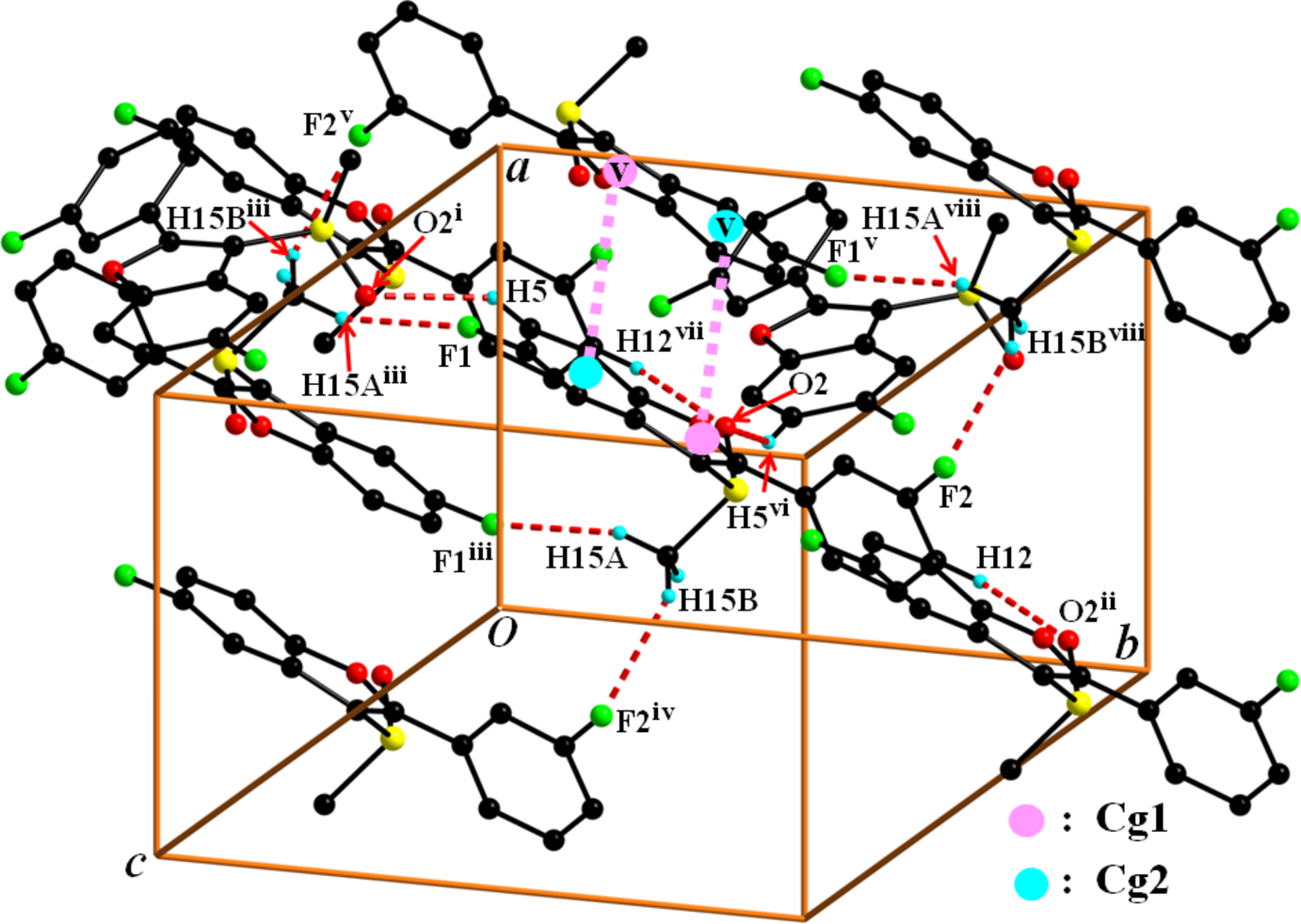

Supplement: Supplementary file 5 [file e-70-o1168-fig2.tif]
